# Anthropometry and the Risk of Breast Cancer in Moroccan Women: A Large Multicentric Case-Control Study

**DOI:** 10.3390/curroncol32080434

**Published:** 2025-07-31

**Authors:** Najia Mane, Najoua Lamchabbek, Siham Mrah, Mohammed Saidi, Chaimaa Elattabi, Elodie Faure, Fatima Zahra El M’rabet, Adil Najdi, Nawfel Mellas, Karima Bendahou, Lahcen Belyamani, Boutayeb Saber, Karima El Rhazi, Chakib Nejjari, Inge Huybrechts, Mohamed Khalis

**Affiliations:** 1 Laboratory of Epidemiology and Research in Health Sciences, Pharmacy and Dental Medicine, Faculty of Medicine, Sidi Mohamed Ben Abdallah University, Fez 30070, Morocco; 2 Department of Public Health and Clinical Research, Mohammed VI Center for Research and Innovation, Rabat 10112, Morocco; 3 Mohammed VI International School of Public Health, Mohammed VI University of Sciences and Health, Casablanca 82403, Morocco; 4 Laboratory Research of Cancer and Chronic Diseases, Faculty of Medicine and Pharmacy of Tangier, Abdelmalek Essaadi University, Tetouan 93000, Morocco; 5 Laboratory “IKLAA” of Law, Philosophy and Society, Sidi Mohamed Ben Abdellah University, Fez 30000, Morocco; 6 International Agency for Research on Cancer, World Health Organization, 69366 Lyon, France; 7 Center of Epidemiology and Population Health, UMR 1018, Inserm, Paris South, Paris Saclay University, 94805 Villejuif, France; 8 Department of Medical Oncology, Hassan II University Hospital of Fez, Fez 30050, Morocco; 9 Department of Epidemiology, Faculty of Medicine and Pharmacy of Casablanca, Hassan II University of Casablanca, Casablanca 9154, Morocco; 10 Department of Medical Oncology, National Institute of Oncology, Rabat 6213, Morocco; 11 Euromed Research Center, Euromed University of Fez, Fez 51, Morocco; 12 French Network for Nutrition and Cancer Research (Nacre Network), 78350 Jouy-en-Josas, France; 13 Higher Institute of Nursing Professions and Health Techniques, Ministry of Health and Social Protection, Rabat 10000, Morocco

**Keywords:** breast cancer, adiposity, anthropometry, obesity, pre- and post-menopausal women, Morocco

## Abstract

Although evidence suggests adiposity as a modifiable risk factor for postmenopausal breast cancer (BC), its association with premenopausal BC remains uncertain. This potential differential relationship for menopausal status has been insufficiently investigated in the Moroccan population due to limited data. This study aims to assess the relationship between various indicators of adiposity and the risk of BC among Moroccan women by menopausal status. A multicenter case-control study was conducted in Morocco between December 2019 and August 2023, including 1400 incident BC cases and 1400 matched controls. Detailed measures of adiposity and self-reported measures from different life stages were collected. Unconditional logistic regression analyses were conducted to estimate odds ratios (ORs) and 95% confidence intervals (95% CIs) for the association between body size indicators and the risk of BC, adjusting for a range of known risk factors for BC. Higher waist circumference (WC) and hip circumference (HC) were associated with an increased risk of BC in both pre- (*p*-trend < 0.001 for both WC and HC) and post-menopausal women (*p*-trend < 0.001 for WC, 0.002 for HC). Current body mass index (BMI) ≥30 kg/m^2^ increased the risk of postmenopausal BC (*p*-trend = 0.012). Among postmenopausal women, higher weight at age 20 was positively associated with BC risk (*p*-trend < 0.001), while, weight at age 30 was significantly associated with increased BC risk in both pre- (*p*-trend = 0.008) and post-menopausal women (*p*-trend = 0.028). Interestingly, weight gain since age 20 was inversely associated with BC risk in postmenopausal women in the adjusted model (*p*-trend = 0.006). Young-adult BMI observed a significant increased trend with BC risk in both pre- (*p*-trend = 0.008) and post-menopausal women (*p*-trend < 0.001). In premenopausal women, larger body shape during childhood and early adulthood was positively associated with BC risk (*p*-trend = 0.01 and = 0.011, respectively). In postmenopausal women, larger childhood and adolescent body silhouettes were also associated with increased BC risk (*p*-trend = 0.045 and 0.047, respectively). These results suggest that anthropometric factors may have different associations with pre- and post-menopausal BC among Moroccan women. This underscores the importance of conducting large prospective studies to better understand these findings and explore their links to different molecular subtypes of BC.

## Simple Summary

Breast cancer is the most common cancer among women worldwide. Although research data suggest a positive association between adiposity and postmenopausal breast cancer, this relationship remains uncertain and inconclusive regarding the risk of premenopausal breast cancer. In the Moroccan population, this relationship remains little studied. This study aims to better understand this relationship by examining several anthropometric measurements and their association with breast cancer by menopausal status. The findings of this study could help identify high-risk groups and integrate targeted prevention strategies adapted to the Moroccan context.

## 1. Introduction

BC is the most common cancer among women worldwide, accounting for 2.3 million new cases, it is the fourth leading cause of cancer-related mortality in the world, responsible for 685,000 deaths, which represents 11.6% of all new cancer fatalities [1]. In developing regions, the incidence of BC is rising. Moreover, nowadays the majority of BC-related deaths occur in low- and middle-income countries (LMICs) [1,2].

BC is the most commonly diagnosed cancer among women in Morocco, and it remains the leading cause of cancer-related mortality in this population. In 2022, it accounted for 38.8% of new cancer cases [3], and led to 4044 deaths among women, representing 25.6% of all cancer-related deaths in women [4]. According to the Casablanca Cancer Registry, BC comprised 22.3% of all cancer cases between 2018 and 2021, with an age-standardized incidence rate (ASIR) of 45.5 per 100.000 women [5]. Like other emerging countries, the rising incidence of BC in Morocco is likely attributed to improvements in screening systems, high exposure to risk factors associated with the adoption of a Western lifestyle, and the effects of globalization, along with social and economic development [1,6].

A variety of factors, including physical inactivity and excess body weight, have been linked to the rising rates of BC [7]. Most studies consistently show that an increased adult body size, as measured by BMI, elevates the risk of postmenopausal BC [8,9,10,11,12], while it appears to be associated with a reduced risk in premenopausal women [10,11,13,14,15]. The results of studies examining the association between BMI and the risk of pre-menopausal BC in African women remain inconsistent, while some studies found no association between BMI and BC risk [16,17], others reported an inverse relationship [18,19]. In post-menopausal African women, some studies have shown that a high BMI is associated with an increased risk of BC [19,20,21].

Numerous studies have shown that the risk of BC increases with central obesity measured by WC in pre- and post-menopausal women [17,22,23]. Additionally, weight gain during adulthood has been found to increase the risk in postmenopausal women [24,25,26]. On the other hand, several studies suggest that a larger body shape in childhood or adolescence are related to a lower risk of BC in both pre- and post-menopausal women [27,28].

Increasing rates of overweight and obesity among women significantly affect health, quality of life, productivity, and healthcare costs, especially in developing countries [29]. In Morocco, obesity and overweight rates are raising among both children and adults of both sexes [30]. A report from the Moroccan High Commission for Planning showed that the prevalence of obesity among Moroccan women increased from 16.0% in 2001 to 29% in 2018 [31]. Similarly, a study found even higher prevalence in 2015 with 38.7% of Moroccan women being overweight and 22.6% obese [32]. In the light of these trends, it seems necessary to examine the link between obesity and the risk of BC in Morocco.

To the best of our knowledge, only one case-control study conducted between 2016–2017, at a single center with 300 cases and 300 controls, has investigated the relationship between body size, body shape trajectory and BC risk among Moroccan women. The study found that higher WC and HC were positively associated with BC risk in both pre- and post-menopausal women, moreover body shape at a younger age (6–11 years) was inversely associated with BC risk in premenopausal women. Women with the greatest increase in body shape trajectory over time had a higher risk of developing BC both before and after menopause [17]. In this study we aim at investigating the relationship between BC risk and various anthropometric measures and shapes across different life stages according to menopausal status in a large multicenter case-control study in Morocco.

## 2. Materials and Methods

### 2.1. Study Design

The present study is a multicentric case-control study (BREAST MOROCCO) conducted among Moroccan women from December 2019 to August 2023. It was carried out in multiple hospitals throughout Morocco, including Ibn Rochd University Hospital in Casablanca, Hassan II University Hospital in Fez, Al Hoceima Oncology Centre, Sheikh Zayed Al Nahyan Oncology Hospital in Tangier, and Al Hassani Provincial Hospital in Nador. A total of 1400 incident cases of BC and 1400 controls participants were recruited for the study.

### 2.2. Study Population

The cases included in the study were women with newly diagnosed BC, either in situ or invasive, histologically confirmed, had not received any treatment and had no prior history of cancer. We recruited all consecutive eligible incident BC cases who presented at the selected hospitals during the study period. Women were considered to be premenopausal if they had reported a menstrual period within the 12 months prior to their BC diagnosis. Postmenopausal women were those who self-reported the absence of menstruation for the past 12 months. The control group was selected randomly from healthy women unrelated to the cases with no history of any cancers, selected from women accompanying or visiting their relatives at the same hospital frequented by the cases. Control women were individually matched to cases by age (±5 years) and place of current residence (urban or rural).

Eligibility criteria for both cases and controls included: participant agrees to participate in the study, is capable of providing informed consent, and is not pregnant or breastfeeding. The participation acceptance rate was 98.2% for cases and 93.8% for controls.

BC family history refers to the presence of one or more cases of BC among a person’s biological relatives or family members, in particular first-degree relatives (mother, sister, daughter) and/or second-degree relatives (maternal or paternal aunt, grandmother).

Family history of cancer is defined as the occurrence of cancer in one or more biological relatives, particularly first-degree relatives (parents, siblings, children) and second-degree relatives (grandparents, aunts, uncles).

### 2.3. Data Collection and Assessment

Data were collected using a structured paper-based questionnaire, administered through in-person interviews conducted by six trained interviewers. After verification of eligibility criteria, patients were invited to participate in the study. The objectives and procedures were explained in detail, and written informed consent was obtained prior to data collection. Anthropometric measurements and interviews were carried out before the start of any treatment, in a dedicated medical consultation room. This standardized recruitment protocol was applied in all five hospitals to ensure consistency. The questionnaire covered a wide range of details, such as socio-demographic characteristics, occupational history, cancer history, reproductive life, smoking history, alcohol consumption, physical activity levels and anthropometric measurements, and dietary intake. Socio-economic status of participants was assessed using a validated wealth score based on household assets including electricity, television, cell phone, refrigerator, indoor bath or shower, indoor tap, flush toilet, washing machine, car and landline phone [33]. The reproducibility and validity of the questionnaire have been assessed in this pilot study.

#### 2.3.1. Physical Activity and Dietary Assessment

To assess physical activity levels, we used a structured questionnaire that captured participants’ engagement in various domains of physical activity, such as occupational, recreational, and household activities. Participants were asked to report the duration of each activity category (low, moderate, and vigorous) for each day of a typical week over the past 12 months. The reported durations for each intensity level were then converted into Metabolic Equivalent Tasks (METs) to estimate overall physical activity levels [34].

Dietary intake over the previous 12 months was assessed using a 255-item Food Frequency Questionnaire (FFQ), that had been previously validated and adapted for use in the Moroccan population, which guaranteed the reliability of the dietary data, as described elsewhere [35].

#### 2.3.2. Body Size and Anthropometrics Measures

Anthropometric measurements were measured for all participants by trained interviewers at the time of recruitment, using standardized methods in accordance with Lohman’s recommendations [36]. Body weight was measured with participants wearing light clothing, using calibrated equipment. Standing height was measured without shoes on a flat, uncarpeted surface using a stadiometer. WC was measured at the natural indentation of the waist, while HC was measured at the widest part of the buttocks. Both WC and HC were recorded with participants in light clothing. The waist-to-hip ratio (WHR) was calculated by dividing WC (in centimeters) by HC (in centimeters). BMI was calculated by dividing weight (in kilograms) by the square of height (in meters). Current BMI was classified according to the World Health Organization (WHO) guidelines into three categories: normal weight (<25 kg/m^2^), overweight (25–29 kg/m^2^), and obese (≥30 kg/m^2^) [37]. To evaluate long-term weight changes, BMI in early adulthood was estimated by dividing the participant’s self-reported weight at age 20 (in kilograms) by the square of their current height (in meters). Weight gain since early adulthood was assessed by calculating the difference between the participant’s current measured weight and their self-reported weight at age 20.

**Figure 1:**
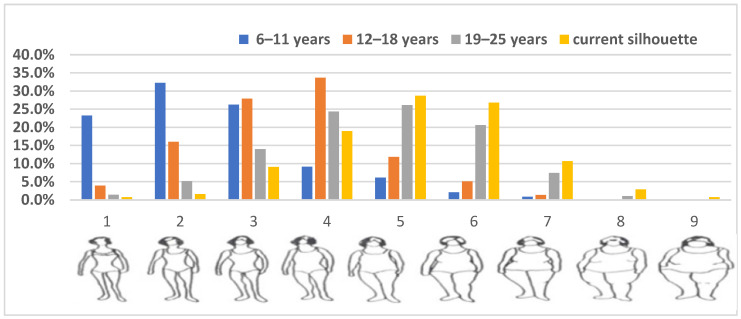
Distribution of perceived body shape silhouettes among Moroccan women across different age groups.

The Stunkard figure rating scale [38], was used to assess body silhouette across different life stages, which includes nine female body shape pictograms ranging from very slim (number 1) to very large (number 9) at six different life stages: childhood (6–11 years), adolescence (12–18 years), early adulthood (19–25 years) and the current body silhouette. Participants were asked to identify the figure that most closely resembled their body shape during each age periods (Figure 1).

To ensure adequate sample sizes and statistical power in analyses silhouette categories, the nine body figures were grouped into broader categories for each life stage. During childhood, figures were categorized as: lean (number 1), medium (number 2), and large (numbers 3–9). For adolescence, the categories were: lean (numbers 1 and 2), medium number 3), and large (numbers 4–9). For early adulthood and current body shape, silhouettes were grouped as: lean (numbers 1–3), medium (number 4), and large (numbers 5–9).

### 2.4. Statistical Analyses

Descriptive statistics were used to compare the key characteristics of the study population between BC cases and controls. Categorical variables were described using frequencies and percentages, and comparisons were performed using chi-square tests. Continuous variables were presented as means with standard deviations (SD) or as medians with interquartile ranges (IQR), depending on the distribution, and compared using independent *t*-tests or the Mann–Whitney U test. To assess the association between body size indicators and the risk of BC, multivariable unconditional logistic regression analyses were conducted. All statistical analyses were run in separate models for premenopausal and postmenopausal women.

The results were expressed as odds ratios (ORs) with corresponding 95% confidence intervals (CIs), accounting for a multiple potential confounding factors, including: age (Years), place of residence, age at menarche (Years), average daily caloric intake (Kcal/day), physical activity (MET min/week), wealth score, educational level (illiterate, elementary/Koranic school, secondary school, high school/Technical or professional school), occupation (housewife, employed, previously employed), history of oral contraceptive use (Yes, No), age at first full-term pregnancy (nulliparous, <22 years, >22 years), breastfeeding duration (never breastfed, >0–<24 months, ≥24 month, nulliparous), family history of BC (Yes, No). Quartiles were constructed based on the distribution of body size indicators among controls within each menopausal group. The two-sided *p*-value < 0.05 was considered statistically significant. All statistical analyses were performed with SPSS version 21.0.

### 2.5. Ethical Consideration

The study protocol was approved by the Research Ethics Committee of the Faculty of Medicine and Pharmacy of Casablanca (No. 09/16) on 3 March 2016. All participants provided written informed consent prior to participation, after being informed about the study’s purpose, confidentiality measures, and their right to withdraw at any time.

## 3. Results

### 3.1. Characteristics of the Study Population

Table 1 presents the overall characteristics of cases and controls, categorized by menopausal status. The study included 1400 cases (634 premenopausal and 765 postmenopausal women) and 1400 controls (649 premenopausal and 749 postmenopausal women). Significant differences were observed between cases and controls across reproductive, lifestyle, and anthropometric characteristics in both pre- and post-menopausal women. Compared to controls, both pre- and post-menopausal women with BC were more likely to have a later age at first full-term pregnancy, a shorter duration of breastfeeding, a family history of cancer and BC, lower levels of moderate physical activity, and a higher BMI, WC, HC and WHR as well as a lower employment rate. Additionally, premenopausal cases had a higher prevalence of illiteracy. In contrast, postmenopausal cases were more likely to have an earlier age at menarche, a later age at menopause, and a greater tendency for abdominal overweight.

### 3.2. Body Size Measures and Body Shape Across Different Life Stages

Table 2 presents the ORs for BC in relation to anthropometric measurements and body shape by menopausal status. Higher WC and HC were significantly associated with an increased risk of BC, with significant trends across quartiles among both pre- (Q4 vs. Q1, OR = 8.41 (5.36–13.18), and OR = 1.74 (1.20–2.53), respectively) and post-menopausal women (Q4 vs. Q1, OR = 3.34 (2.37–4.71), and OR = 1.48 (1.07–2.04), respectively). Furthermore, current BMI was significantly positively associated with BC risk only among postmenopausal women (Q4 vs. Q1, OR = 1.75 (1.25–2.46). While, young-adult BMI showed a significant increased trend with BC risk in both pre- (Q4 vs. Q1, OR = 1.84 (1.29–2.62) and post-menopausal women (Q4 vs. Q1, OR = 3.39 (2.44–4.71)). Nevertheless, no significant association was found between current weight and BC risk in either pre- or post-menopausal women.

The multivariable -adjusted ORs comparing the highest to the lowest quartile of weight at the age of 20 showed a significant increasing trend in BC risk among postmenopausal women (Q4 vs. Q1, OR = 1.83 (1.34–2.51)). However, no such trend was observed among premenopausal women (*p*-trend = 0.122). Whereas, a significant increasing BC risk observed with increased weight at the age of 30 among both premenopausal (Q4 vs. Q1, OR = 1.45 (1.01–2.08)), and postmenopausal women (Q4 vs. Q1, OR = 1.83 (1.34–2.51)). Further, weight gain since the age of 20 was not significantly associated with premenopausal BC in either the crude or adjusted models. Similarly, no significant association was found with postmenopausal BC in the crude model. However, in the adjusted model, a significant decreasing trend was observed when comparing the highest quartile (≥15 kg) to the lowest quartile (<5 kg) (Q4 vs. Q1, OR = 0.64 (0.47–0.87)).

When comparing larger versus leaner silhouettes among premenopausal women, a significant positive association was observed between BC risk and larger body silhouette during childhood (6–11 years) (OR = 1.71 (1.22–2.38)), and early adulthood (19–25 years) (OR = 1.31 (0.94–1.84)). However, no significant association was found between BC risk and body silhouette during adolescence (12–18 years) or the current silhouette. For postmenopausal women, a significant increased trend was observed between BC risk and childhood silhouette (6–11 years) (OR = 1.40 (1.05–1.88)), adolescent silhouette (12–18 years) (OR = 1.29 (0.96–1.75)), and current silhouette (OR = 1.17 (0.80–1.73)). However, no significant association was found between BC risk and early adulthood silhouette (19–25 years). In addition, the reported distribution of excess weight—whether in the chest, shoulders, thighs, or whole body, showed no significant association with either pre- or postmenopausal BC, except for abdominal obesity, which was significantly associated with increased risk (OR = 1.71 (1.05–2.80)).

## 4. Discussion

BC is the leading cause of morbidity and mortality among women in developing countries, including Morocco. Determining modifiable risk factors for BC is essential for developing evidence-based prevention strategies that are of benefit to public health.

This large case-control study of Moroccan women, found a positive association between WC, HC and both pre- and post-menopausal BC. Furthermore, current BMI was significantly positively associated with BC risk only among postmenopausal women. A significant association between BMI in young adulthood and BC risk was observed in both groups. Additionally, weight at age 20 was positively associated with postmenopausal BC, while weight at age 30 was significantly linked to pre-and postmenopausal BC. Finally, we observed that larger body size during childhood and early adulthood was associated with an increased risk of BC in premenopausal women, while in postmenopausal women, the increased risk was linked to larger body size during childhood, adolescence, and at the current age.

### 4.1. Overall Adiposity

Previous studies have indicated that higher BMI is associated with a decreased risk of BC in premenopausal women [11,13]. However, a recent meta-analysis involving 22,362 premenopausal Asian women found a higher BC risk with increased BMI [39]. Similar to our study, some studies on premenopausal African women found no association between BMI and BC risk [16,17]. While others African studies reported an inverse relationship between higher BMI and BC risk for premenopausal women [19]. These differing findings may be due to various factors, such as genetic or environmental influences on obesity-related BC pathogenesis [40], or estrogen-related factors, as women with higher BMIs tend to have longer, more irregular menstrual cycles with increased anovulation, leading to lower estrogen and progesterone levels [41].

Consistent findings from the literature show a significant positive association between high BMI and an increased risk of postmenopausal BC [8,11,12], which is also observed in our study. The main theory in this association is that obesity leads to the aromatization of adrenal androgens into estrogen in adipocytes [8,42,43], which is often linked to estrogen receptor (ER)-positive BC in overweight/obese women, but not ER-negative BC [8,42]. Further supporting the hormone theory is the attenuation of the association between BMI and BC risk in women with a history of postmenopausal therapy treatment [8]. Other theories suggest that insulin resistance and increased insulin-like growth factor-1 (IGF-1) promote cell proliferation and higher free estrogen levels through a reduction in sex-hormone-binding globulin [43]. While differences in fat distribution before and after menopause may also impact breast tissue [44].

**Table 1:** Characteristics of population cases and controls according to menopausal status, 2019–2023.

Characteristics	Premenopausal Women (*n* = 1283)			Postmenopausal Women (*n* = 1514)		
	Controls (*n* = 649)	Cases (*n* = 634)	*p* Value	Controls (*n* = 749)	Cases (*n* = 765)	*p* Value
Age (years)	42.00 (10)	42.00 (10)	0.83	58 (9)	59 (10)	0.39
Area of residence			0.443			0.43
Urban	425 (65.5)	428 (67.5)		528 (70.5)	525 (68.6)	
Rural	224 (34.5)	206 (32.5)		221 (29.5)	240 (31.4)	
Marital status			0.02			<0.001
Single	173 (26.7)	128 (20.2)		81 (10.8)	116 (15.2)	
Married	430 (66.3)	449 (70.8)		524 (70.0)	474 (62.0)	
Divorced	36 (5.5)	38 (6.0)		51 (6.8)	50 (6.5)	
Widowed	10 (1.5)	19 (3.0)		93 (12.4)	125 (16.3)	
Occupation			<0.001			<0.001
Housewife	444 (68.4)	419 (66.1)		586 (78.2)	548 (71.6)	
Employed	164 (25.3)	107 (16.9)		79 (10.5)	59 (7.7)	
Previously employed	41 (6.3)	108 (17.0)		84 (11.2)	158 (20.7)	
Educational level			<0.001			0.14
Illiterate	209 (32.2)	302 (47.6)		452 (60.3)	504 (65.9)	
Elementary/Koranic school	137 (21.1)	157 (24.8)		159 (21.2)	146 (19.1)	
Secondary school	187 (28.8)	136 (21.5)		105 (14.0)	85 (11.1)	
High school/Technical or professional school	116 (17.9)	39 (6.2)		33 (4.4)	30 (3.9)	
Wealth score	12 (3)	13 (2)	<0.001	13 (2)	13 (2)	0.02
Family history of cancer			<0.001			<0.001
Yes	110 (16.9)	206 (32.5)		154 (20.6)	216 (28.2)	
No	539 (83.1)	428 (67.5)		595 (79.4)	549 (71.8)	
Family history of breast cancer			<0.001			<0.001
Yes	32 (4.9)	84 (13.2)		44 (5.9)	101 (13.2)	
No	617 (95.1)	550 (86.8)		705 (94.1)	664 (86.8)	
Age at menarche (years)	13 (2)	13 (1)	0.06	12 (2)	12.5 (1)	<0.001
Age at full-term pregnancy (years) ‡	22 (5)	23 (6)	0.004	20 (6)	22 (7)	<0.001
Parity			0.12			0.07
Parous	438 (67.5)	453 (71.5)		597 (79.7)	580 (75.8)	
Nulliparous	211 (32.5)	181 (28.5)		152 (20.3)	185 (24.2)	
History of breastfeeding			<0.001			<0.001
Never Breastfeed	9 (2.1)	17 (3.7)		5 (0.8)	27 (4.7)	
>0–<24 months	65 (15.0)	147 (32.3)		83 (13.9)	140 (24.4)	
≥24 months	359 (82.9)	291 (64.0)		509 (85.3)	406 (70.9)	
History of oral contraceptive			<0.001			<0.001
Yes	307 (47.3)	390 (61.5)		337 (45.0)	445 (58.2)	
No	342 (52.7)	244 (38.5)		412 (55.0)	320 (41.8)	
Age at menopause (years)				50 (4)	51 (5)	<0.001
Alcohol						
Yes	4 (0.6)	5 (0.8)		5 (0.7)	1 (0.1)	
No	635 (99.4)	629 (99.2)		724 (99.3)	764 (99.9)	
Moderate physical activity (MET h/week)	63.5 (72)	49.5 (63)	<0.001	63 (63)	45 (63)	<0.001
Energy Intake (Kcal/day)	2536.3 (1197.2)	2592.3 (772.4)	0.43	2385.8 (1040.4)	2411.8 (945.2)	0.56
Weight (kg)	70 (9)	70 (11)	0.41	70 (10)	71 (13)	0.07
Height (cm)	164 (6)	162 (6)	<0.001	163 (6)	161 (7)	<0.001
Current BMI	26.5 (3.2)	27.1 (4.3)	0.002	26.6 (3.29)	27.4 (4.9)	<0.001
Waist circumference (cm)	91 (17)	91 (17)	<0.001	98 (19)	104 (13)	<0.001
Hip circumference (cm)	111 (15)	116 (12)	<0.001	113 (16)	116 (12)	<0.001
Waist-hip ratio	0.85 (0.14)	0.87 (0.06)	<0.001	0.87 (0.14)	0.91 (0.08)	<0.001
Overweight Zone			0.29			<0.001
Chest and shoulders	40 (6.2)	33 (5.2)		67 (8.9)	55 (7.2)	
Thighs	66 (10.2)	81 (12.8)		95 (12.7)	72 (9.4)	
Belly	112 (17.3)	127 (20.0)		125 (16.7)	215 (28.1)	
Whole body	386 (59.5)	348 (54.9)		412 (55.0)	369 (48.2)	
Maintain the sameweight	45 (6.9)	45 (7.1)		50 (6.7)	54 (7.1)	

MET, metabolic equivalents: BMI, Body mass index. ‡ Among parous women.

**Table 2:** Association between anthropometric measurements and risk of breast cancer in Moroccan women by menopausal status using unconditional logistic regression analyses.

		Premenopausal Women (*n* = 1283)					Postmenopausal Women (*n* = 1514)					
		Co/Ca	OR Crude	*p* Trend	OR ^a^ (95% CI)	*p* Trend		Co/Ca	OR Crude	*p* Trend	OR ^a^ (95% CI)	*p* Trend
Current BMI (kg/m^2^)				0.096		0.866				0.007		0.012
	<25 kg/m^2^	224/224	1		1		<25 kg/m^2^	243/254	1		1	
	25–29 kg/m^2^	341/296	0.87 (0.68–1.11)		0.76 (0.57–1.00)		25–29 kg/m^2^	392/336	0.82 (0.65–1.03)		0.82 (0.64–1.06)	
	≥30 kg/m^2^	73/114	1.56 (1.10–2.21)		1.13 (0.75–1.80)		≥30 kg/m^2^	96/175	1.74 (1.29–2.37)		1.75 (1.25–2.46)	
BMI Quartile (kg/m^2^)										0.002		0.015
	Q1 < 24.69	157/165	1	0.003	1	0.65	Q1 < 24.68	183/193	1		1	
	Q2 [24.69–26.12]	162/95	0.71 (0.53–0.96)		0.50 (0.34–0.76)		Q2 [24.68–26.18]	183/139	0.67 (0.51–0.88)		0.79 (0.57–1.09)	
	Q3 [26.12–27.80]	160/140	0.40 (0.29–0.55)		0.68 (0.47–0.98)		Q3 [26.18–28.13]	185/149	0.48 (0.36–0.64)		0.78 (0.56–1.08)	
	Q4 ≥ 27.80	159/234	0.60 (0.44–0.81)		0.97 (0.68–1.37)		Q4 ≥ 28.13	180/284	0.51 (0.38–0.68)		1.43 (1.05–1.95)	
Weight (kg)				0.072		0.951				0.67		0.945
	Q1 < 66	179/201	1		1		Q1 < 65	202/259	1		1	
	Q2 [66–70]	182/123	0.60 (0.44–0.82)		0.46 (0.32–0.65)		Q2 [65–70]	218/163	0.58 (0.44–0.77)		0.56 (0.41–0.76)	
	Q3 [70–75]	145/116	0.71 (0.52–0.98)		0.52 (0.36–0.76)		Q3 [70–76]	153/128	0.65 (0.48–0.88)		0.59 (0.43–0.83)	
	Q4 ≥ 75	132/194	1.31 (0.97–1.77)		0.97 (0.68–1.38)		Q4 ≥ 76	158/215	1.06 (0.81–1.40)		1.02 (0.75–1.38)	
Waist circumference (cm)				<0.001		<0.001				<0.001		<0.001
	Q1 < 84	171/42	1		1		Q1 < 87	199/96	1		1	
	Q2 [84–91]	158/100	2.58 (1.69–3.92)		2.51 (1.57–4.01)		Q2 [87–95]	186/101	1.13 (0.80–1.59)		1.11 (0.77–1.62)	
	Q3 [91–101]	162/190	4.76 (3.21–7.10)		5.18 (3.30–8.14)		Q3 [95–105]	186/273	3.04 (2.24–4.13)		2.81 (2.00–3.93)	
	Q4 ≥ 101	158/302	7.78 (5.28–11.48)		8.41 (5.36–13.18)		Q4 ≥ 105	178/295	3.44 (2.53–4.67)		3.34 (2.37–4.71)	
Hip circumference (cm)				<0.001		<0.001				<0.001		0.002
	Q1 < 103	166/114	1		1		Q1 < 102	187/155	1		1	
	Q2 [103–111]	159/90	0.82 (0.58–1.17)		0.63 (0.42–0.94)		Q2 [102–112]	220/173	0.95 (0.71–1.30)		0.89 (0.65–1.22)	
	Q3 [111–118]	163/212	1.89 (1.38–2.59)		1.20 (0.83–1.74)		Q3 [112–118]	158/205	1.57 (1.16–2.11)		1.34 (0.96–1.88)	
	Q4 ≥ 118	161/218	1.97 (1.44–2.70)		1.74 (1.20–2.53)		Q4 ≥ 118	184/232	1.52 (1.14–2.03)		1.48 (1.07–2.04)	
Weight at the age of 20 (kg)				0.675		0.122				0.012		<0.001
	Q1 < 56	172/168	1		1		Q1 < 55	194/177	1		1	
	Q2 [56–60]	205/213	1.06 (0.80–1.42)		1.12 (0.81–1.57)		Q2 [55–60]	287/288	1.10 (0.85–1.43)		1.14 (0.85–1.53)	
	Q3 [60–65]	135/90	0.68 (0.49–0.96)		0.86 (0.58–1.27)		Q3 [60–64]	106/72	0.74 (0.52–1.07)		0.93 (0.62–1.37)	
	Q4 ≥ 65	137/163	1.22 (0.89–1.66)		1.45 (1.01–2.08)		Q4 ≥ 64	162/228	1.54 (1.16–2.05)		1.83 (1.34–2.51)	
Weight at the age of 30 (kg)				<0.001		0.008				0.07		0.028
	Q1 <65	178/139	1		1		Q1 < 60	192/172	1		1	
	Q2 [65–70]	210/157	0.96 (0.71–1.30)		0.98 (0.69–1.40)		Q2 [60–68]	199/190	1.07 (0.80–1.42)		1.14 (0.85–1.53)	
	Q3 [70–77]	106/144	1.74 (1.25–2.43)		1.59 (1.07–2.35)		Q3 [68–75]	192/217	1.26 (0.95–1.67)		0.93 (0.62–1.37)	
	Q4 ≥ 77	155/194	1.60 (1.18–2.18)		1.45 (1.01–2.08)		Q4 ≥ 75	166/186	1.25 (0.93–1.68)		1.83 (1.34–2.51)	
Weight gain since the age of 20 (kg)				0.434		0.068				0.178		0.006
	Q1 < 5	179/236	1		1		Q1 < 5	196/281	1		1	
	Q2 [5–10]	202/104	0.39 (0.29–0.53)		0.36 (0.26–0.52)		Q2 [5–10]	196/135	0.48 (0.36–0.64)		0.42 (0.31–0.58)	
	Q3 [10–14]	106/91	0.65 (0.46–0.92)		0.50 (0.33–0.74)		Q3 [10–15]	158/141	0.62 (0.47–0.83)		0.49 (0.35–0.68)	
	Q4 ≥ 14	151/203	1.02 (0.77–1.36)		0.69 (0.49–0.98)		Q4 ≥ 15	181/208	0.80 (0.61–1.05)		0.64 (0.47–0.87)	
Young-adult BMI (at age 20 (kg/m^2^))				0.027		0.008				<0.001		<0.001
	Q1 < 20.96	167/139			1		Q1 < 20.81	186/116	1		1	
	Q2 [29.96–22.58]	150/166	1.33 (0.97–1.82)		1.42 (0.99–2.05)		Q2 [20.81–22.50]	189/178	1.51 (1.11–2.06)		1.504 (1.06–2.12)	
	Q3 [22.58–24.22]	171/112	0.79 (0.57–1.09)		0.90 (0.62–1.31)		Q3 [22.50–24.10]	187/156	1.34 (0.98–1.83)		1.63 (1.14–2.31)	
	Q4 ≥ 24.22	160/217	1.63 (1.20–2.21)		1.84 (1.29–2.62)		Q4 ≥ 24.10	185/315	2.73 (2.03–3.67)		3.39 (2.44–4.71)	
Body size at 6–11 years				<0.001		0.01				0.009		0.045
	Lean	193/105	1		1			189/141	1		1	
	Medium	183/231	2.32 (1.71–3.15)		1.74 (1.23–2.48)			203/229	1.51 (1.13–2.02)		1.44 (1.05–1.98)	
	Large	273/298	2.01 (1.50–2.68)		1.71 (1.22–2.38)			357/395	1.48 (1.14–1.93)		1.40 (1.05–1.88)	
Body size at 12–18 years				0.013		0.28				0.016		0.047
	Lean	145/110	1		1			153/135	1		1	
	Medium	184/174	1.25 (0.90–1.72)		0.95 (0.65–1.38)			213/186	0.99 (0.73–1.34)		1.00 (0.72–1.39)	
	Large	320/350	1.44 (1.08–1.93)		1.15 (0.82–1.62)			383/444	1.31 (1.00–1.72)		1.29 (0.96–1.75)	
Body size at 19–25 years				<0.001		0.011				0.033		0.097
	Lean	143/120	1		1			163/157	1		1	
	Medium	200/112	0.67 (0.48–0.93)		0.59 (0.40–0.87)			204/160	0.81 (0.60–1.10)		0.84 (0.60–1.16)	
	Large	306/402	1.57 (1.18–2.08)		1.31 (0.94–1.84)			382/448	1.22 (0.94–1.58)		1.20 (0.90–1.61)	
Current body size				0.017		0.333				0.004		0.048
	Lean	80/65	1		1			70/66	1		1	
	Medium	139/104	0.92 (0.61–1.39)		0.86 (0.53–1.38)			173/115	0.71 (0.47–1.06)		0.76 (0.49–1.18)	
	Large	430/465	1.33 (0.94–1.89)		1.10 (0.72–1.67)			506/584	1.22 (0.86–1.75)		1.17 (0.80–1.73)	
Overweight Zone				*p* value					*p* value		*p* value	
	Maintain the same weight	40/33	1					67/55	1		1	
	Chest and shoulders	66/81	1.49 (0.85–2.62)	0.168	0.79 (0.39–1.62)	0.521		95/72	0.76 (0.45–1.28)	0.305	0.89 (0.50–1.58)	0.696
	Thighs	112/127	1.37 (0.81–2.33)	0.236	0.95 (0.51–1.74)	0.859		125/215	0.70 (0.43–1.15)	0.158	0.74 (0.44–1.27)	0.277
	Belly	386/348	1.09 (0.67–1.77)	0.719	1.1 (0.62–1.95)	0.738		412/369	1.59 (1.02–2.48)	0.04	1.71 (1.05–2.80)	0.031
	Whole body	45/45	1.21 (0.65–2.25)	0.542	0.79 (0.62–1.95)	0.356		50/54	0.83 (0.55–1.25)	0.37	0.94 (0.60–1.48)	0.8

Abbreviations: BMI = body mass index; CI = Confidence interval; OR = odds ratio; Q = Quartile. All values are OR (95% CI). a: Adjusted for age (Years), place of residence, age at menarche (Years), average daily caloric intake (Kcal/day), physical activity (MET min/week), wealth score, educational level (illiterate, elementary/Koranic school, secondary school, high school/Technical or professional school), occupation (housewife, employed, previously employed), history of oral contraceptive use (Yes, No), age at first full-term pregnancy (nulliparous, <22 years, >22 years), breastfeeding duration (never breastfed, >0–<24 months, ≥24 month, nulliparous), family history of breast cancer (Yes, No).

Moreover, we found a positive association between early adulthood BMI (at age 20) and both pre- and post-menopausal BC, consistent with a Tanzanian case-control study [16]. However, a pooled analysis of 20 cohort studies reported an inverse association between early adulthood BMI and both pre- and post-menopausal BC [15]. Adiposity in early life stages, could impact the way fat is deposited during adulthood, with later weight gain influencing hormonal changes, notably estrogen, which play a significant role in BC development [26]. In addition, it has recently been recognized that early body fat influences BC risk by modifying breast density, which is a known risk factor for BC [26,45]. The AMBER Consortium study of premenopausal African American women found an inverse relationship between higher BMI at age 18 and ER+ BC risk, without any association to ER- or triple-negative BC [46]. This association may vary according to the molecular subtypes of BC [46,47], highlighting the need for further longitudinal studies with hormonal information to examine the underlying biological mechanisms in Moroccan women.

Our findings align with previous studies showing that adult weight gain (since age 20) is not linked to premenopausal BC [24,25,26,47,48]. However, a recent pooled study of 17 prospective cohort studies from the Premenopausal Breast Cancer Collaborative Group found that weight gain between ages 18–24 and 35–54 was inversely associated with premenopausal BC [49]. On the contrary, weight gain during adulthood has been reported in many studies to be associated with an increased risk of postmenopausal BC [24,25,26,48], which differs from the lower risk found in our study.

The mechanism linking BC and weight gain is not fully understood. Endogenous hormone levels and mammographic breast density may play a role [47]. It is possible that weight gain reflects an indicator of hormonal environment, and that the same factors leading to increased body weight may also increase BC risk. Additionally, energy balance at certain life stages could play a crucial role [48]. Evidence suggests that the timing of weight gain, body fat distribution, and tumor receptor status may also affect this relationship [25,48]. Preventing weight gain in adulthood could potentially decrease postmenopausal BC risk [50]. Longitudinal studies with detailed anthropometric data are needed to better understand the link between body size throughout life and BC risk in Moroccan women.

### 4.2. Central Adiposity

A number of previous studies, including the present one, have shown a positive association between higher WC, HC and BC risk in both pre- and post-menopausal women [17,22]. A recent meta-analysis including 57 studies (26 case-control and 31 cohort) found that central obesity, measured by WC, increased the risk of both pre- and post-menopausal BC [23]. However, a multicentric population-based case-control study identified a negative correlation between adult adiposity (WC, HC) and BC risk [51]. These contrasting associations are complex to interpret. The strong link between WC and high insulin levels [52] may explain the relationship between WC and increased BC risk [53], while specific properties of gluteal adipose tissue, indicated by HC [54] such as leptin secretion, may contribute to the association with HC. Additionally, since WC and HC are only indicators of body fat distribution, techniques like DEXA (dual-energy X-ray absorptiometry) or impedance, which provide more details on body composition and fat distribution, could help clarify these associations [51].

Reverse causality, which refers to the possibility that changes in body composition related to the disease or its treatment might have influenced WC or HC for pre- and post-menopausal subjects, and thereby distorted the observed associations, is unlikely in this study. Anthropometric measurements were taken at the time of the first consultation with the oncologist, prior to the initiation of any treatment (chemotherapy, hormone therapy, or radiotherapy). Moreover, a recent study conducted in 2022 on the prevalence of overweight and obesity in Morocco reported high rates of general and particularly abdominal obesity among Moroccan women [55].

This supports the likelihood that the high central adiposity observed in our study reflects background population characteristics rather than disease-related changes. Furthermore, the associations observed between central adiposity and breast cancer risk are consistent with the findings of Khalis et al., conducted in a Moroccan population [17], supporting the validity of our results.

### 4.3. Body Shape Across Different Stages

Our results show a positive association between self-reported body silhouette during childhood and early adulthood and the risk of premenopausal BC. This contrasts with a case- control study among Latin American women, which found no association between body shape at younger ages and premenopausal BC risk [51], and with results of a previous case-control study in Moroccan women [17]. In Mexico, another case-control study found a strong inverse association between a larger body shape at ages 18–20 and premenopausal BC risk [56]. Other studies have linked larger body sizes between ages 7–18 with a reduced risk of both premenopausal and postmenopausal BC [28,57]. In postmenopausal women, childhood and adolescent body shape and current body size were significantly associated with an increased BC risk, though no significant link was found between body shape in early adulthood and BC risk. These results contrast with a Mexican study, which reported a significant inverse association between larger body shape in childhood and BC risk [56].

These results may be explained by the varying influence of early-life body size on breast cancer risk across different populations [51], or they could suggest that the silhouette scales, though validated in European [58] and North American [59] populations, may not be fully suited for our population. However, we cannot entirely rule out the possibility of recall bias, as women may have misremembered their body size at different ages. Collecting somatotype information many years later could lead to individuals reporting a more consistent body size pattern and less variation over time.

### 4.4. Strengths and Limitations

Our study is the largest multicentric case-control study examining the association between BC risk and anthropometric measurements and shapes according menopausal status in Moroccan women. Our cases are new patients whose diagnosis of BC has been confirmed by pathologists, which reduces classification and information bias. It is also the first study in Morocco investigating associations between BC risk and these body shapes at different time points throughout the lifecycle. In addition, a large battery of socio-demographical and lifestyle data were collected, allowing adjustments for potential confounding in the statistical models. Some limitations of our study should be noted. Potential errors or biases in relation to the self-reported measurements should be considered. In particular, random errors linked to retrospective memory are likely to occur, such as when participants are asked to indicate their weight at the age of 20. Our study may be subject to recall bias particularly social desirability bias, that may arise in studies examining associations between disease and height/weight using self-reported measures, as overweight individuals often underestimate their weight, while shorter or underweight individuals may overestimate it [60]. This type of bias was minimized by training the interviewers.

### 4.5. Implications and Recommendations

It is essential to follow the World Cancer Research Fund (WCRF) guidelines which mainly focus on obesity related lifestyle factors [61], and the WHO guidelines on reducing obesity [62], as well as its recent guidelines on physical activity [63], and nutrition [64]. It is also important to assessing the interventions needed at national level from a normative and cultural point of view. Therefore, weight management is a key to decreasing BC risk, alongside controlling food intake and ensuring adequate physical activity. Policy interventions targeting the obesogenic environment should encompass multiple areas, including the food industry and urban planning, in order to promote access to healthy food choices and opportunities for physical activity. Public awareness campaigns promoting healthy lifestyles should be integrated into educational institutions [13].

This study underscores the need for further longitudinal research to better clarify the complex and potentially causal relationship between obesity and BC risk according to menopausal status. To this end, large-scale prospective cohort studies are essential to minimize bias due to reverse causation and to examine the succession over time of changes in weight and the onset of BC.

Obesity is an important risk factor for BC, particularly in post-menopausal women. Clinicians should systematically assess their patients’ weight and BMI as part of their cancer risk assessment. Early detection of overweight or obese patients means that lifestyle changes, particularly in terms of diet and physical activity, can be recommended in time to reduce the risk of cancer. Integrating weight management programs into basic healthcare can improve general health and potentially reduce the incidence of breast cancer. In addition, healthcare professionals should make patients aware of the link between obesity and breast cancer in order to encourage them to adopt preventive practices.

## 5. Conclusions

In our study, we identified several statistically significant associations with pre- and post-menopausal BC, including WC, HC and BMI at age 20. Notably, higher current BMI and weight at age 20 increased the risk of postmenopausal BC, while weight at age 30 was significantly associated with increased risk of pre-and postmenopausal BC. We also found a positive association between body shape at childhood and pre-and post-menopausal BC. Our results confirm and support the conclusions of previous research which suggest that avoiding weight gain throughout life is a way of reducing the risk of BC, particularly in the postmenopausal period. These findings underscore the importance of focusing on primary prevention strategies. Therefore, Moroccan women should adopt healthier lifestyles through healthy nutrition and regular exercise to reduce the risk of developing BC. In addition, early detection and regular screening are proactive approaches for detecting BC and for reducing the risk of BC mortality.

## Data Availability

The data used and/or analyzed in this study may be supplied by the corresponding author, subject to a justified request.

## Abbreviations

The following abbreviations are used in this manuscript:

ASIR	Age-standardized incidence rate
BC	Breast cancer
BMI	Body mass index
CIs	Confidence intervals
DEXA	Dual-energy X-ray absorptiometry
ER	Estrogen receptor
FFQ	Food Frequency Questionnaire
HC	Hip circumference
IGF-1	Insulin-like growth factor-1
IQR	Interquartile ranges
LMICs	Low- and middle-income countries
METs	Metabolic Equivalent Tasks
OR	Odd ratios
Q	Quartile
SD	standard deviations
WC	Waist circumference
WCRF	World Cancer Research Fund
WHO	World Health Organization
WHR	Waist hip ratio
